# Congenital adrenal hyperplasia with homozygous and heterozygous mutations: a rare family case report

**DOI:** 10.1186/s12902-022-00969-w

**Published:** 2022-03-07

**Authors:** Tiantian Cheng, Jing Liu, Wenwen Sun, Guangyao Song, Huijuan Ma

**Affiliations:** 1 Department of Internal Medicine, School of Clinical Medicine, North China University of Science and Technology, Tangshan, 063210 Hebei China; 2 Department of Endocrinology and Metabolic Diseases, Hebei General Hospital, Shijiazhuang, 050051 Hebei China; 3 Hebei Key Laboratory of Metabolic Diseases, Hebei General Hospital, Shijiazhuang, 050051 Hebei China

**Keywords:** Congenital adrenal hyperplasia (CAH), Steroid 21-hydroxylase deficiency;p.I173N,c.1451-1452delGGinsC, Case report

## Abstract

**Background:**

Congenital adrenal hyperplasia (CAH), characterized by defective adrenal steroidogenesis, is transmitted in an autosomal recessive manner. Mutations in the steroid 21-hydroxylase gene *CYP21A2* causing steroid 21-hydroxylase deficiency account for most cases of CAH. The c.145l-1452delGGinsC gene mutation is rare, and only one case has been reported, but the form of gene mutation is different from this case, resulting in different clinical phenotype. The most common pathogenic genotype of CAH is a homozygous or compound heterozygous mutation, but CAH patients homozygous for the p.I173N mutation and heterozygous for the c.1451-1452delGGinsC mutation have not been reported previously. We report herein a familial case of CAH, in which both siblings carry the rare homozygous p.I173N mutation and heterozygous c.1451-1452delGGinsC mutation.

**Case presentation:**

The proband showed amenorrhea, infertility, polycystic ovaries, and increased levels of androgen, rather than the typical clinical manifestations of CAH such as an adrenal crisis or masculine vulva, so was misdiagnosed with polycystic ovary syndrome for many years. Following a correct diagnosis of CAH, she was given glucocorticoid treatment, her menstruation became more regular, and she became pregnant and delivered a healthy baby girl.

**Conclusions:**

The genotypes may be p.I173N homozygous or p.I173N/c.1451-1452delGGinsC heterozygous, both mutations could be pathogenic. This complex combination of mutations has not been reported or studied before. Through the report and analysis of this genotype, the content of CAH gene bank is enriched and the misdiagnosis rate of CAH is reduced.

## Background

Congenital adrenal hyperplasia (CAH) is a rare autosomal recessive disease [1]. Its pathogenesis is characterized by a defect in adrenal steroidogenesis caused by a mutation in one or more enzyme-encoding genes, leading to dysfunctional cortisol and aldosterone production and excessive levels of androgen [2]. Steroid 21-hydroxylase deficiency (21-OHD) caused by mutations in the *CYP21A2* gene located on the short arm of chromosome 6 [3] accounts for more than 90% of CAH cases [4].CAH resulting from 21-hydroxylase deficiency can be classified into two clinical forms according to the complete deficiency and partial deficiency of enzyme activity: classical (either salt-wasting or simple virilization) and non-classical [5]. Compared with salt-wasting CAH, patients with simple virilizing or non-classical CAH produce an part insufficient amount of cortisol leading to slight symptoms of hypocortisolism, the clinical symptoms and signs caused by hyperandrogenism are more prominent [6]; thus it is common for such women to be misdiagnosed with polycystic ovary syndrome.

A previous study showed that the micro-conversion of p.I173N accounted for 14.3% of *CYP21A2* micro-conversions in CAH [7]. However, pathogenic variants are often compound heterozygous, with the homozygous c.518T>A (p.I173N) mutation being only rarely reported [8], and the homozygous c.518T>A (p.I173N) mutation with the heterozygous c.1451-1452delGGinsC (p.R484Pfs*58) mutation not documented. Here, we describe a familial case of CAH in which both siblings carry the homozygous p.I173N mutation and the heterozygous p.R484Pfs*58 mutation. Genetic analysis showed that both parents of the siblings carry the same heterozygous p.I173N mutation, while the mother also carries the heterozygous p.R484Pfs*58 mutation. The proband had been misdiagnosed with polycystic ovary syndrome for many years, but after our diagnosis of CAH she was given glucocorticoid treatment, and eventually became pregnant and gave birth to a healthy baby girl.

## Case presentation

### Patient 1

The 31-year-old female proband had experienced amenorrhea for 7 years. Oligomenorrhoea and hypomenorrhoea are fairly common in CAH. She had not used contraception since her marriage 6 years ago and had not become pregnant during this time. She repeatedly sought medical advice, and was diagnosed with polycystic ovary syndrome after a gynecological ultrasound examination revealed polycystic ovaries, and a hormone test showed elevated testosterone (T) levels of 2.94 ng/ml. She was prescribed a range of medication, including metformin, spironolactone, dydrogesterone, and herbal medicine after which she had a normal menstrual cycle for a short time.

She attended the outpatient clinic of our hospital, and presented with amenorrhea and infertility. Serological examination revealed elevated adrenocorticotropic hormone (ACTH) levels of 357.8 pg/ml (normal, 7.2–63.3 pg/ml), luteinizing hormone (LH) levels of 2.52 mIU/ml (normal, 2.4–12.6 mIU/ml), follicle stimulating hormone (FSH) levels of 3.40 mIU/ml (normal, 3.85–8.78 mIU/ml), prolactin levels of 28.91 ng/ml (normal, 6–29.9 ng/ml), estradiol levels of 73.80 ng/ml (normal, 12.4–233 ng/ml), progesterone (P) levels >40 ng/ml (normal, 0.2–1.5 ng/ml), and T levels of 4.03 ng/ml (normal, 0.06–0.82 ng/ml). She was hospitalized for further treatment and received an outpatient diagnosis of hyperandrogenism, polycystic ovary syndrome.

**Fig. 1:**
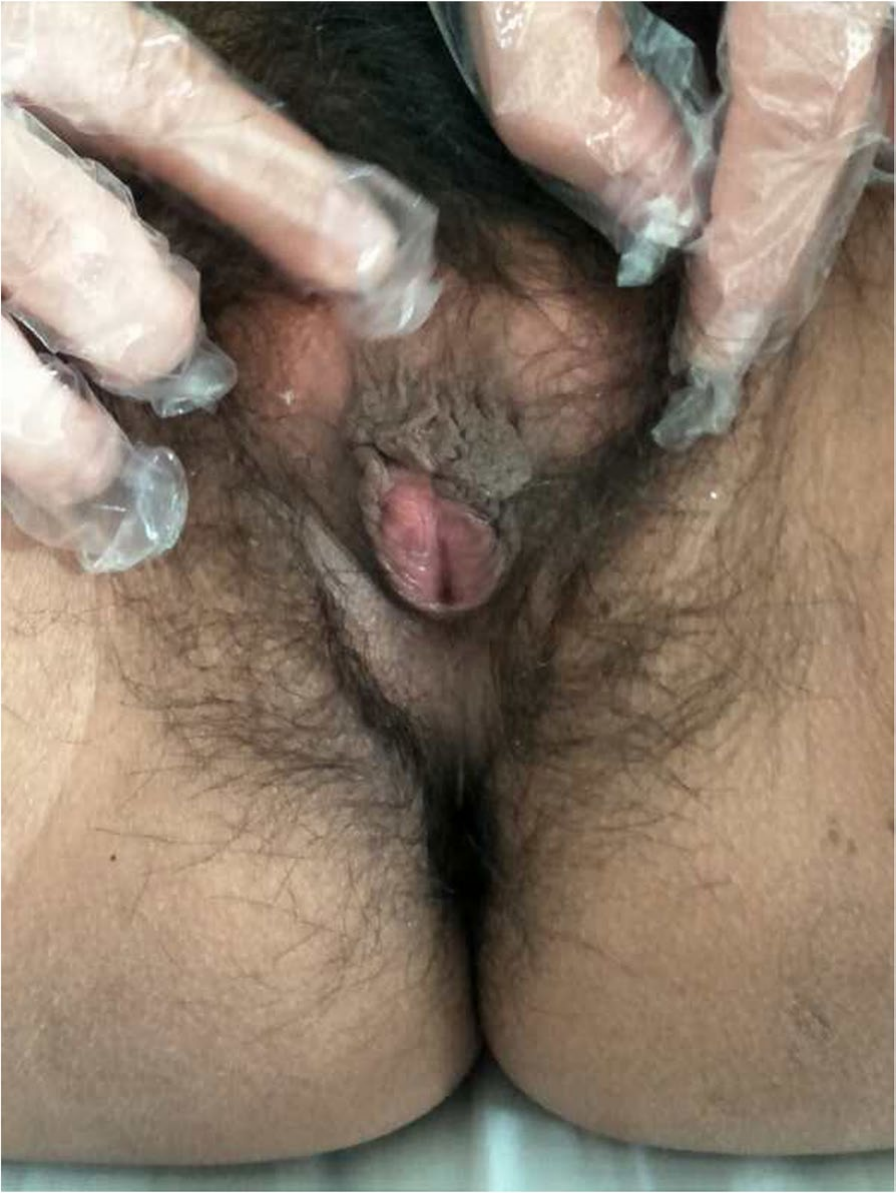
The vulva of the proband showing cliteromegaly

Her menarche occurred at the age of 14, with an initial menstrual cycle of 28–30 days and periods lasting 5–7 days; at this time, menstruation was moderate with no dysmenorrhea. Menstruation began to decrease at the age of 20, with occasional menstrual cramps and a small volume of blood loss; amenorrhea gradually occurred from the age of 24. She married at the age of 25 and was not pregnant at this time. Her height is 149 cm, her father’s height is 168 cm, her mother’s height is 164 cm, and her younger brother’s height is 160 cm. Her parents have no corresponding clinical manifestations of CAH. Her borther is divorced and childless. On physical examination, her blood pressure was 124/65 mmHg. She is thin, with no full moon face or buffalo hump, rough skin, a dark complexion, and no acne. Her lower abdomen and lower limbs have thick hair, her vulvar hair is heavy and pigmented, and clitoromegaly was evident (Fig. 1).

**Fig. 2:**
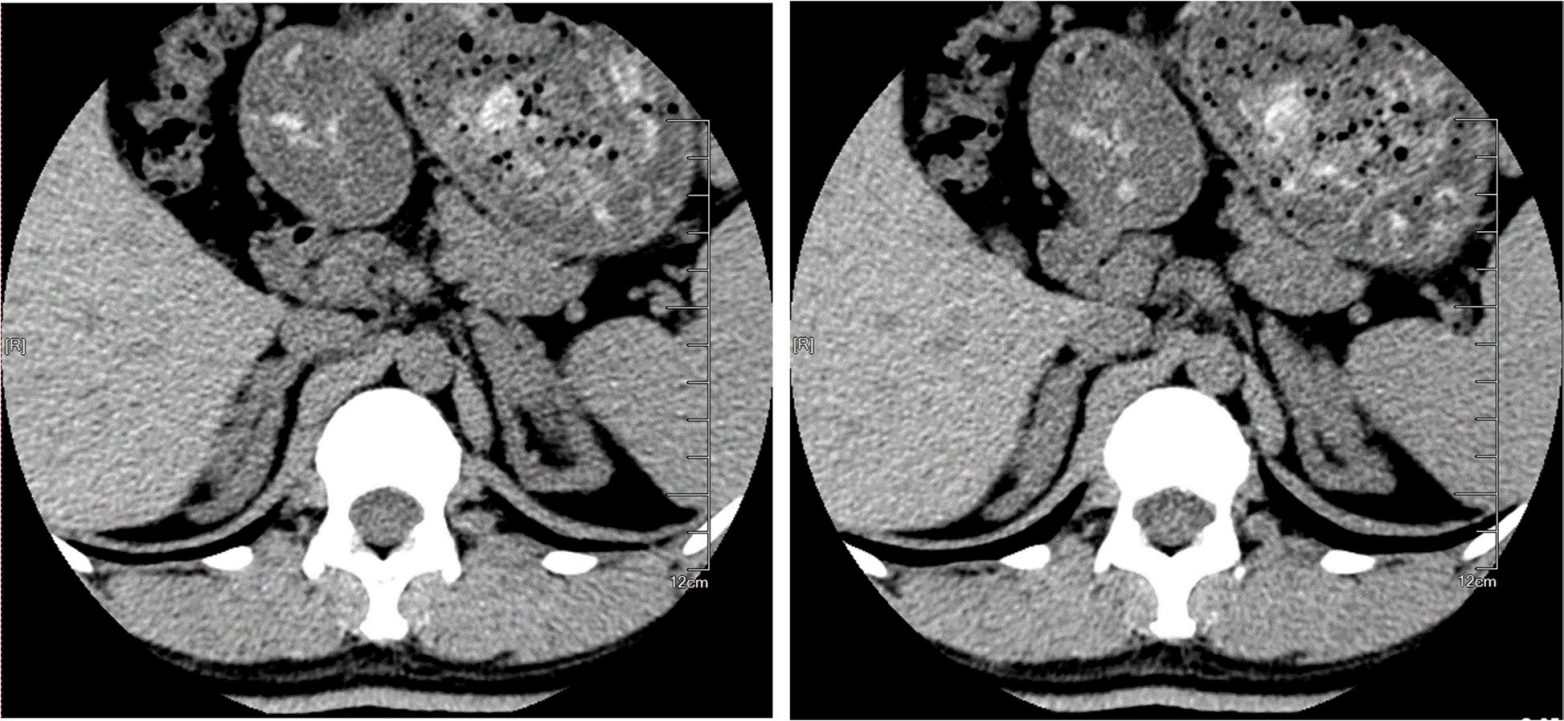
Adrenal computed tomography of the proband showing an increased bilateral adrenal volume

Laboratory examination revealed normal levels of electrolytes: potassium 3.9 mmol/L (normal, 3.5–5.3 mmol/L), and sodium 141 mmol/L (normal, 137–147 mmol/L). Serum cortisol (F) rhythms (00:00 h–08:00 h–16:00 h) were 3.24, 13.72, and 13.12 μg/dl (normal morning, 6.2–19.4 μg/dl; afternoon 2.3–11.9 μg/dl). ACTH rhythms were 313.5, 384.3, and 222.3 pg/ml (normal, 7.2–63.3 pg/ml), and 24 h urinary free cortisol levels were 316.16 μg/24 h (normal, 30–350 μg/24 h).17-hydroxyprogesterone (17-OHP) levels were 182.8 ng/ml which was much higher than normal (0.05–1.02 ng/ml). Androstenedione (AD) levels were >10 ng/ml (normal, 0.30–3.30 ng/ml), dehydroepiandrosterone (DHEA) was 4.12 ng/ml (normal, 0.80–10.50 ng/ml), and T was 4.03 ng/ml (normal, 0.06–0.82 ng/ml) (Table 1). A glucose tolerance test showed that blood sugar levels were normal, but an insulin release test revealed that the peak of insulin secretion exceeded the basic value by 10 times (Table 2). Adrenal computed tomography showed an increased bilateral adrenal volume with nodular processes (Fig. 2).

**Table 1:** Patient laboratory test results

	17-OHP (ng/ml)	AD (ng/ml)	DHEAS (ng/ml)	T (ng/ml)	LH (mIU/ml)	FSH (mIU/ml)	E2 (pg/ml)	P (ng/ml)	F (ug/dl)	ACTH (pg/ml)	PRL (ng/ml)	Electrolyte	
												Na (mmol/l)	K (mmol/l)
**Before treatment**	182.8	>10	4.12	4.03	2.52	3.40	73.8	>40	13.72	384.3	28.91	141	3.9
**Treatment for 2 months**	2.64	-	-	0.16	1.21	3.54	157.7	1.05	0.084	3.98	34.23	142	3.8
**Treatment for 7 months**	0.86	-	-	<0.025	7.86	4.12	239.00	2.70	<0.054	3.51	20.63	138	3.9
**Treatment for 13 months**	0.98	-	-	<0.025	4.01	4.04	37.77	4.60	0.167	26.4	41.05	140	4.1
**Normal ranges**	0.05-1.02	0.30-3.30	0.80-10.50	0.06-0.82	2.4-12.6	3.85-8.78	12.4-233	0.2-1.5	6.2-19.4	7.2-63.3	6-29.9	137-147	3.5-5.3

**Table 2:** Glucose tolerance test results of the proband

	0min	30min	60min	120min	180min
blood glucose (mmol/L)	4.95	8.88	10.68	5.94	6.17
insulin (uU/ml)	7.14	82.26	133.1	65.64	64.91
C peptide (ng/ml)	2.14	8.39	13.74	11.75	9.47

Gynecological ultrasound showed the uterus to be in the anteversion position, 39 × 26 × 27 mm in size, with an endometrial thickness of 4.2 mm, and a normally shaped cervix. The left ovary was 29 × 21 mm in size, and seven follicles were seen on a single section with a maximum diameter of about 6 mm. The right ovary was 35 × 14 mm, and 10 follicles were seen on a single section with a maximum diameter of about 5 mm. Magnetic resonance imaging of the pituitary showed that the pituitary stalk was positioned slightly to the right. Her karyotype was 46,XX. According to the gene test result, she was shown to carry the *CYP21A2* mutation (as described below). Her final diagnosis was 21-hydroxylase deficiency CAH.

### Patient 2

Her 30-year-old brother attended our hospital because of infertility after marriage. On physical examination his skin was normal in color, his genitalia were normal, and his height was 160 cm. Laboratory examination revealed normal F levels of 9.46 μg/dl (normal, 6.02–19.4 μg/dl), but ACTH levels of 166.5 pg/ml (normal, 7.2–63.3 pg/ml), FSH <0.1 mIU/ml (normal, 1.27–19.2 mIU/ml), LH of 0.33 mIU/ml (normal, 1.7–8.6 mIU/ml), 17-OHP >300 ng/ml (normal, 0.31–2.01 ng/ml), AD >10 ng/ml (normal, 0.6–3.1), DHEA of 19.06 ng/ml (normal, 3.35–13.8 ng/ml), and T of 12.2 ng/ml (normal, 2.8–8 ng/ml) (Table 3). His karyotype was 46,XY. According to the gene test result, he was shown to carry the *CYP21A2* mutation (as described below). He was also diagnosed with 21-hydroxylase deficiency CAH.

**Table 3:** Laboratory test results of patient's brother

	17-OHP (ng/ml)	AD (ng/ml)	DHEAS (ng/ml)	T (ng/ml)	LH (mIU/ml)	FSH (mIU/ml)	F (ug/dl)	ACTH (pg/ml)
**Before treatment**	208.03	>10	19.06	12.2	<0.1	0.33	9.46	166.5
**Treatment for 10 months**	-	-	-	5.94	<0.1	0.58	9.03	134.8
**Treatment for 11 months**	-	-	-	5.85	<0.1	0.45	10.86	103.1
**Normal ranges**	0.31-2.01	0.6-3.1	3.35-13.8	2.8-8	1.7-8.6	1.27-19.2	6.02-18.4	7.2-63.3

**Fig. 3:**
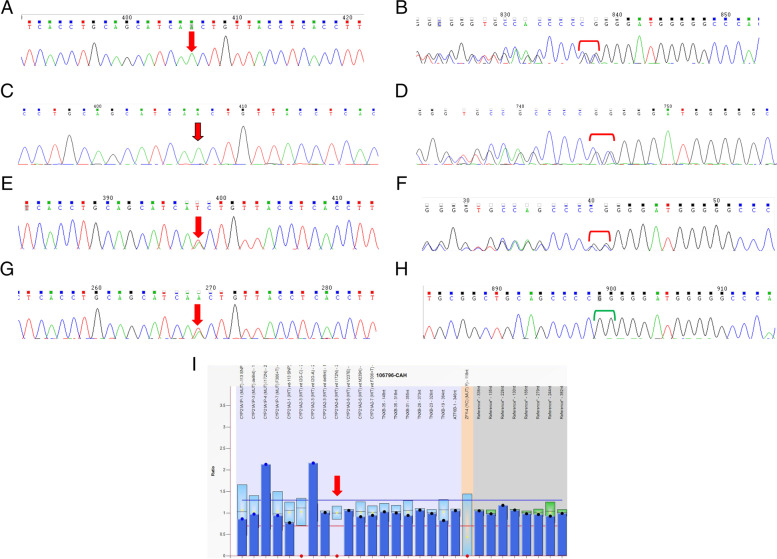
Mutation sites of the siblings and their family. **a** Homozygous c.518T>A (p.I173N) CYP21A2 mutation seen in the proband. **b** Heterozygous c.1451-1452delGGinsC (p.R484pfs*58) CYP21A2 mutation seen in the proband. **c** Homozygous c.518T>A (p.I173N) CYP21A2 mutation seen in the proband’s brother. **d** Heterozygous c.1451-1452delGGinsC (p.R484pfs*58) CYP21A2 mutation seen in the proband’s brother. **e** Heterozygous c.518T>A (p.I173N) CYP21A2 mutation of the proband’s mother. **f** Heterozygous c.1451-1452delGGinsC (p.R484pfs*58) CYP21A2 mutation of the proband’s mother. g. Heterozygous c.518T>A (p.I173N) CYP21A2 mutation of the proband’s father. **h** Normal sequence at this CYP21A2 locus in the proband’s father. **i** MLPA results showing that ZFY4 was a Y chromosome probe, so its absence in females is normal

**Fig. 4:**
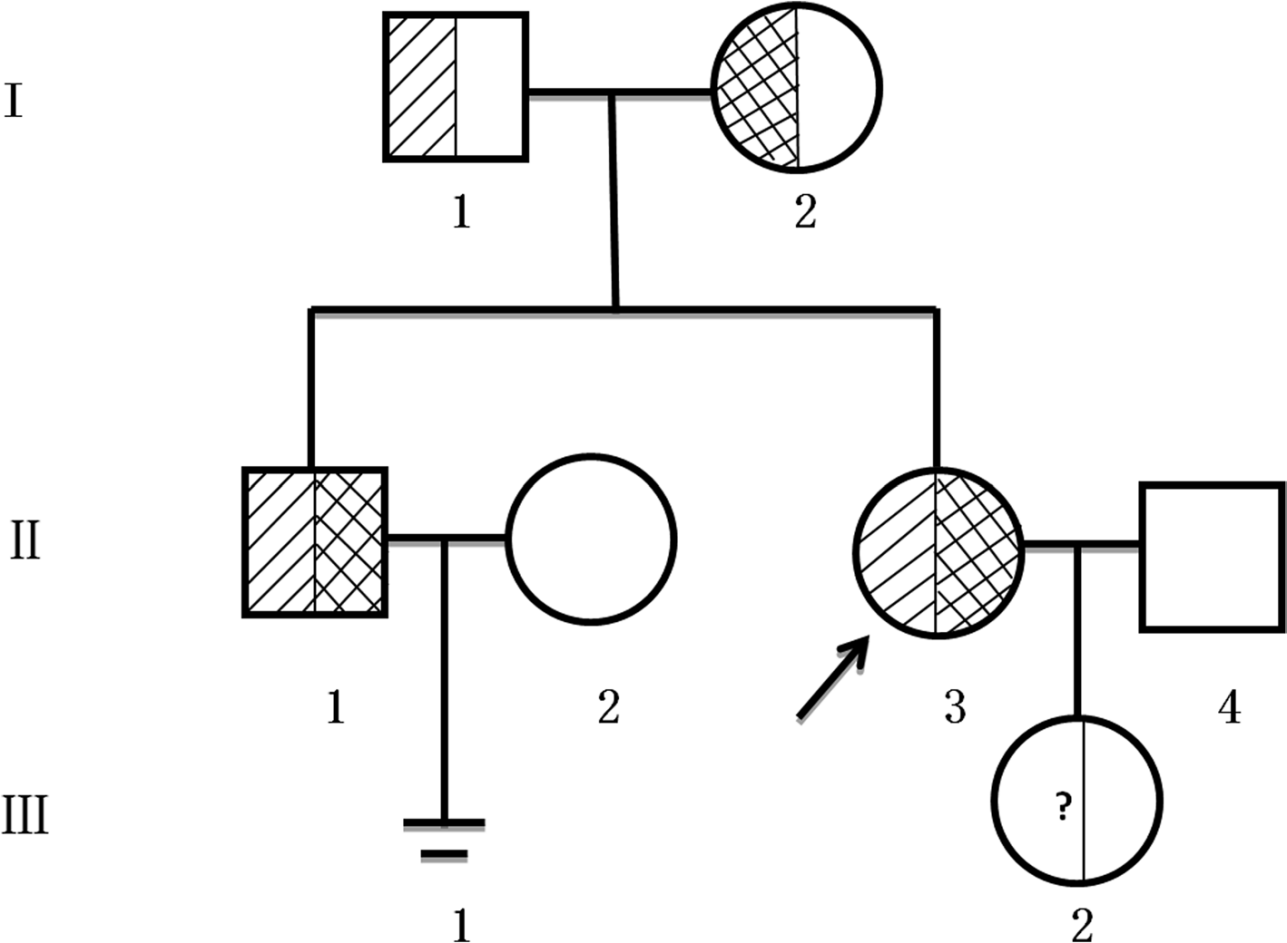
Family pedigree. I-1 is the proband’s father, I-2 is her mother, II-1 is her brother, II-2 is her brother’s spouse, II-3 is the proband, and II-4 is her spouse. III-1 indicates that the proband’s brother is childless, and III-2 is the proband’s daughter

With the informed consent of both patients and their families, we extracted 2 ml of peripheral blood from the proband, her brother, and their parents. Genomic DNA was isolated from peripheral blood leukocytes using the DNA QIAamp mini kit (Qiagen, Hilden, Germany), according to the manufacturer's instructions. Exons of the proband were captured using the BGI-Exome kit and whole exome sequencing was performed for 100 bp paired-end reads using the BGI-seq 2000 platform. Low-quality reads were removed by SOAPnuke, then remaining reads were mapped to the human genome (reference UCSCGRCh37/hg19) by Burrows–Wheeler Aligner software (BWA-MEM, version 0.7.10). The Genome Analysis Tool Kit (GATK, version 3.3) was used to call the variants, which were then annotated and classified by ANNOVAR software. Our in-house exome data interpretation pipeline was used to select prior candidates. Pathogenicity prediction tools including SIFT (http://provean.jcvi.org/), PolyPhen2 (http://genetics.bwh.harvard.edu/pph2/), and MutationTaster (http://www.mutationtaster.org/) were used to predict the functional impact of candidate variants. The wild-type and variant-type protein structure was predicted by I-TASSER and the two models were compared with Pymol. DNA from all four family members underwent Sanger sequencing to validate the variants and confirm their co-segregation. PCR products amplified with primers designed by Primer3 software were sequenced on an ABI 3730XL DNA Analyzer. The father was shown to carry the heterozygous *CYP21A2* c.518T>A (p.I173N) mutation, and the mother also carried this mutation and the heterozygous c.1451-1452delGGinsC (p.R484Pfs*58) mutation. Both siblings carried the homozygous c.518T>A (p.I173N) and heterozygous c.1451-1452delGGinsC (p.R484Pfs*58) mutations (Table 4, Fig 3). According to the results of genetic diagnosis, one chromosome of father carry the heterozygous CYP21A2 c.518T>A (p.I173N) mutation, and one chromosome of mother carried this mutation and the heterozygous c.1451-1452delGGinsC (p.R484Pfs*58) mutation, while both chromosomes of patient and her younger brother carry mutation genes (Fig 4).

**Table 4:** Genetic sequencing results of the proband and her brother

Nucleotide changes	Gene subregion	Homozygous/heterozygous	Amino acid change	Pathogenicity analysis	Mutation source
c.518T>A	CDS4	homozygous	p.I173N	pathogenicity	patrents
c.1451-1452delGGinsC	CDS10	heterozygous	p.R484Pfs*58	possibly pathogenicity	mother

Sibling sequencing results are identical

The proband was given dexamethasone 0.75 mg every night after her diagnosis. Menstruation occurred after 1 month of medication, with a moderate flow, and a period length of about 4 days. Two months later, 17-OHP was greatly reduced at 2.64 ng/ml (normal, 0.05–1.02 ng/ml), and T was 0.16 ng/ml (normal, 0.06–0.82 ng/ml). Dexamethasone was therefore reduced to 0.375 mg every night, after which T, F, and ACTH levels were relatively stable. She was thereafter treated with dexamethasone 0.375 mg every night. During this treatment, her menstrual cycle was about 28 days, with a moderate flow, and a menstrual period of 4–6 days. After 13 months of treatment, she became pregnant by artificial insemination, and continued dexamethasone 0.375 mg during this period. In February 2020, she gave birth to a healthy baby girl, of normal height and weight and with a normal vulva. She continued to be treated with dexamethasone after delivery, and 17-OHP and androgen levels were within normal ranges upon regular reexamination (Table 1).

The proband’s younger brother also received treatment after his sister was diagnosed with CAH. Dexamethasone at 0.75 mg each night was given initially, and after 10 months T levels had fallen to 5.94 ng/ml (normal, 2.8–8 ng/ml, F was 9.03 μg/dl (normal, 6.02–19.4 μg/dl), and ACTH was 134.8 pg/ml (normal, 7.2–63.3 pg/ml). Dexamethasone was then adjusted to 0.375 mg per night, and after 11 months of treatment the levels of T, F, and ACTH remained stable (Table 3). Both siblings received dexamethasone 0.375 mg / day as early replacement therapy, and were regularly followed up.

## Discussions and conclusions

Mutational defects in the steroid 21-hydroxylase gene *CYP21A2* causing steroid 21-hydroxylase deficiency account for over 90% of CAH cases [9]. Most genetic defects result from misalignments between the gene and the pseudogene during meiosis that result in deletions, large gene conversions, duplications, or the presence of more than one pathogenic variant in the same allele [10]. To date, more than 100 *CYP21A2* mutations have been reported that are associated either with severe salt-wasting or simple virilizing phenotypes or with milder nonclassical phenotypes [11].

Wide phenotypic variability is observed in simple virilizing CAH, particularly in those cases caused by the I173N mutation in exon 4 of *CYP21A2* [12]. Normal transcription has been reported to be associated with some I2G mutations, resulting in limited enzyme activity [12]. Another study suggested that extraadrenal 21-hydroxylase activity affects the clinical phenotype, with liver CYP2C19 and CYP3A4 enzymes shown to be active against progesterone, and to regulate mineralocorticoid deficiency to some extent. Moreover, multiple genes are thought to regulate 21-hydroxylase deficiency [13].

The deletion mutation c.145l-1452delGGinsC is located on exon 10 of *CYP21A2*, and is a frameshift mutation at arginine 484. The predicted mutated protein is 45 amino acids longer than the normal one, and is likely to result in enzyme inhibition. Wedell et al. reported a 3-week-old patient carrying the compound heterozygous mutation p.R357w/c.1454-1452delGGinsC, with the classic salt-wasting CAH phenotype [14]. The clinical phenotype of patients with 21-hydroxylase deficiency is often thought to be determined by the mutation that results in less damage to enzyme activity. Although p.R357w has been associated with the complete loss of enzyme activity [15], this patient had a salt-wasting phenotype, so it is speculated that c.1454-1452delGGinsC can cause complete loss of 21-hydroxylase activity.

In our familial cases of CAH, the father of the proband carried the p.I173N mutation, while the mother carried both p.I173N and c.1451-1452delGGinsC mutations. It was therefore unusual for both siblings to carry p.I173N on one chromosome and c.1451-1452delGGinsC on the other. Both mutations could be pathogenic, and the sibling genotypes may be p.I173N homozygous or p.I173N/c.1451-1452delGGinsC heterozygous. Because this complex combination of mutations has not been reported or studied before, we cannot determine which plays a leading role in disease pathogenesis or whether they jointly cause disease.

Both siblings were diagnosed with simple virilizing CAH. Previous work showed that the homozygous p.I173N mutation can lead to partial enzyme deficiency, resulting in simple virilizing CAH [16]. Additionally, c.1451-1452delGGinsC was reported to cause severe salt-wasting CAH [14]. However, the characteristics of autosomal recessive disorders are such that the clinical phenotype caused by a mild mutation can resemble that caused by a severe mutation. Therefore, the partial enzyme activity caused by the p.I173N mutation covers the serious clinical phenotype caused by c.1451-1452delGGinsC, so the resulting phenotype can also be characterized as simple virilizing CAH. Therefore, in these cases the clinical phenotype is a simply virilizing CAH determined by the p.I173N mutation that cause a 1-2% of residual enzyme activity. Because this complex pathogenic genotype has not been reported previously, and is consistent with the proband’s clinical phenotype, it is not clear whether one mutation plays a leading role in pathogenesis or if their effects are equal. This is relevant to other mutation-related research, so should be explored further.

The discovery of this complex mutation combination also plays a vital role in future genetic inheritance. The proband’s daughter had a normal birth length, weight, and vulva, which is considered to reflect the absence of pathogenic mutations inherited from her father. However, it is conceivable that she may pass on two pathogenic mutations to her own children, increasing the chance that the next generation will suffer from severe classical CAH. Therefore, we recommend that the proband undergoes genetic counselling before attempting to become pregnant again.

Patients with simple virilizing CAH show mild cortisol deficiency and androgen excess, with clitoromegaly (100%) and skin hyperpigmentation (87%) reported as the most common features [6]. Hirsutism, oligomenorrhea or amenorrhea, and decreased fertility are also common symptoms [17]. Salt wasting and adrenal crises only occur in an emergency state, so patients often experience long delays before being correctly diagnosed and treated [18]. The first medical visit is usually made because of amenorrhea, infertility, high T levels, or polycystic ovary changes detected by gynecological ultrasound, which can be misdiagnosed as polycystic ovary syndrome. Patients may be transferred to several hospitals and ineffectively treated, resulting in women of childbearing age being infertile. Such women should be alert to the possibility of CAH, with the main diagnostic criteria including increased 17-hydroxyprogesterone and androstenedione and decreased cortisol levels [19]. Indeed, a diagnosis of polycystic ovary syndrome can be excluded when 17-hydroxyprogesterone levels exceed 10 ng/ml, or even 5.4 ng/ml [20]. Moreover, women with simple virilizing CAH have a higher prevalence of normal ovulation and a lower likelihood of an LH/FSH ratio >2 or polycystic ovaries compared with those with polycystic ovary syndrome. Additionally, most polycystic ovary syndrome patients have insulin resistance [21].

Gynecological ultrasound of our proband revealed many small follicles with a diameter less than 10 mm on both ovaries, an LH/FSH ratio >2, and high T levels, which were suggestive of polycystic ovary syndrome, and made an accurate diagnosis more difficult. However, the examination of 17-hydroxyprogesterone, androstenedione, cortisol, and ACTH levels, and genetic analysis enabled an accurate diagnosis of CAH to be made. Men with CAH have a high risk of developing hypothalamic–pituitary–gonadal disturbances and spermatogenic abnormalities [22]. Testicular adrenal rest tumors, oligospermia, and hypogonadotropic hypogonadism are also frequently associated with subfertility in men with all forms of CAH [23], which could explain the infertility seen in the proband’s brother.

The main treatment for all forms of CAH is glucocorticoid replacement therapy, such as dexamethasone, prednisone, methylprednisolone, hydrocortisone and fludrocortisone. In addition, in recent years, a new treatment Chronocort, a modified-release hydrocortisone formulation has been gradually applied to adult patients with CAH [24]. Dexamethasone is generally used for adults who stop growing. This may benefit adult women with fertility problems, hirsutism, and other skin symptoms. It can also reduce the time taken for women with CAH to become pregnant, and will not increase the abortion rate in early pregnancy [25]. Dexamethasone is used to prevent prenatal virilization in female fetuses with CAH [26]. Most pregnant women carrying an identified 46,XX CAH fetus who go ahead with PreDex therapy at an early stage of gestation (before 6 weeks) take it until delivery. However, its use before delivery is controversial because it has been reported to reduce fetal weight and increase the intrauterine growth retardation rate in a dose-dependent manner [27]. Other glucocorticoids may also have similar adverse consequences, with hydrocortisone and fludrocortisone shown to be negatively associated with growth velocity [28]. To prevent the premature masculinization of the fetus *in utero*, we chose to continue dexamethasone treatment of the proband before and during pregnancy. Follow-up showed that her menstruation became regular after medication, and that she became pregnant and gave birth to a healthy girl, albeit prematurely at 30 weeks’ gestation.

In conclusion, CAH is a rare autosomal recessive disease that is readily misdiagnosed as polycystic ovary syndrome in patients with simple virilizing or nonclassical CAH whose external genitalia are not obviously masculinized. Therefore, CAH should be excluded from suspected polycystic ovary syndrome patients who are not obese and have elevated T levels. Genetic analysis is also important in a diagnosis, and can be used to screen other family members to enable a timely diagnosis to be made. Genetic testing can also guide fertility, and appropriate prenatal and postnatal care can reduce the economic burden of families and society.

## Data Availability

All data generated or analysed during this study are included in this published article.

## Abbreviations

CAH: Congenital adrenal hyperplasia
T: Testosterone
ACTH: Adrenocorticotropic hormone
LH: Luteinizing hormone
FSH: Stimulating hormone
P: Progesterone
F: Serum cortisol
17-OHP: 17-hydroxyprogesterone
AD: Androstenedione
DHEA: Dehydroepiandrosterone
